# High-Throughput Sequencing Reveals the Loss-of-Function Mutations in *GALT* Cause Recessive Classical Galactosemia

**DOI:** 10.3389/fped.2020.00443

**Published:** 2020-08-05

**Authors:** Lulu Li, Li Ma, Min Sun, Jiancheng Jiao, Yudong Zhang, Yue Tang, Nan Yang, Yuanyuan Kong

**Affiliations:** ^1^Department of Newborn Screening Center, Beijing Obstetrics and Gynecology Hospital, Capital Medical University, Beijing Maternal and Child Health Care Hospital, Beijing, China; ^2^Department of Neonatology, Hebei Provincial Children's Hospital, Shijiazhuang, China

**Keywords:** classical galactosemia (CG), high-throughput sequencing, *GATL*, gene mutation, hepatomegaly, autosomal recessive (AR), Chinese patient, mutation spectrum

## Abstract

**Background:** Classical Galactosemia (CG) is a rare autosomal recessive metabolic disease caused by mutations in the galactose-1-phosphate uridyl transferase (*GALT*) gene. This study aim to identify pathogenic mutations underlying classic galactosemia in two Chinese families.

**Methods:** We collected blood samples from two Chinese families and extracted genomic DNA. High-throughput sequencing, sanger sequencing, and bioinformatics analysis were used to investigate the molecular cause of manifestations in the two Chinese families.

**Results:** We found compound heterozygous mutations (c.396C>G; p.His132Gln and c.974C>T; p.Pro325Leu) in family 1 and a homozygous missense variant (c.974C>T; p.Pro325Leu) in family 2. Bioinformatics and Sanger sequencing were performed to verify the identified variants.

**Conclusion:** The present study identified the *GALT* mutations as a genetic etiology in the two Chinese families with classic galactosemia and expanded the phenotypic and mutational spectrum of *GALT*. Our findings could be useful in providing evidence for prenatal interventions and more precise pharmacological treatments to patients. High-throughput sequencing conducted in our study is a convenient and useful tool for clinical diagnosis of galactosemia and other associated genetic disorders.

## Introduction

Classical Galactosemia (CG) (Type I galactosemia OMIM # 230400) is a rare autosomal recessive inborn error of galactose metabolism, It is caused by galactose-1-phosphate uridylyltransferase (GALT, EC 2.7.7.12) enzyme deficiency (1, 2). The action of the GALT enzyme is to convert galactose-1-phosphate and uridine diphosphate glucose into glucose-1-phosphate and uridine diphosphate-galactose (3). The incidence rates of CG has been reported as 1/16–1/60,000 individuals in various global populations (4, 5). Clinical features associated with CG are feeding difficulty, diarrhea, jaundice, liver and renal complications, muscular hypotonia, cataract, and low intelligence level (1, 6). CG disease caused by the failure to metabolize galactose is potentially life-threatening. With the benefit of early diagnosis by newborn screening, the acute presentation of CG can be prevented. The advent of high-throughput sequencing technology in the field of genetics has provided an unprecedented opportunity for the identification of rare pathogenic variants causing Mendelian disorders.

In this article, we demonstrate two Chinese families with hallmark features of CG. By high throughput sequencing technology we identified novel compound heterozygous and homozygous mutations in the *GALT* gene in family 1 and 2, respectively.

## Methods and Materials

### Family Recruitment and Ethical Sight

This study was approved by the Ethics Committee of Beijing Obstetrics and Gynecology Hospital affiliated to Capital Medical University, and family members (or guardians) all signed informed consent.

### Blood Samples Collection and DNA Extraction

Blood sample was drawn from the affected and normal individuals. Genomic DNA was extracted using phenol chloroform method and was quantified using Nanodrop-2000 by standard methods.

### High-Throughput Sequencing

One to three micrograms of genomic DNA from each sample was sheared into fragments of about 200 bp using the Bioruptor NGS sonication device (Diagenode, Seraing, Belgium). The fragments were purified with Agencourt AMPure XP Kit (Beckman Coulter, Indianapolis, IN). An adenine base was added to the end-repaired DNA fragments followed by ligation to paired-end adapters, amplification for the adapter-ligated library, and quality examination of the amplified library. Nimblegen SeqCap EZ Exome Plus Kit (Roche, Basel, Switzerland) was utilized to hybridize the sample and SeqCap EZ libraries. The capture beads were prepared using Invitrogen Dynabeads M-280 Streptavidin (Thermo Fisher Scientific) and were washed after DNA binding. Captured DNA was amplified via ligation-mediated PCR before purification of the amplified captured multiplex DNA sample. After inspection of reading quality, the captured library was subject to sequencing on the HiSeq 2500 System (Illumina, San Diego, CA).

### Bioinformatics Analysis

Sequencing data was analyzed, filtered, and compared with the human genome reference sequence hg19 (GRCh37/hg19). To identify plausible pathogenic mutations, we mainly focused on non-synonymous homozygous or compound heterozygous variants with a minor allele frequency of 1% (dbSNP142 or ExAC) were retained. The variants were cross-checked in the Human Gene Mutation Database (HGMD; http://www.hgmd.cf.ac.uk/ac/index.php) to see if the identified variants are novel or already reported.

### Primer Designing and Mutation Confirmation

Primer 5.0 primer software was used to design the specific PCR primers (*GALT*-exon 5-F/R: 5′-GTAGCACAGCCAAGCCCTAC-3′/5′-CCCAGAACCAAAGCTTCATC-3′; *GALT*-exon10-F/R: 5′-CAGATACCTGGTTGGGTTTG-3′/5′-GACGCCAGACTGTTCTGAGT-3′). Target region was amplified by polymerase chain reaction (PCR) machine (Takara*/*Clontech). The PCR reaction was commenced with an initial 3-min denaturation step at 95°C, followed by 38 cycles of denaturation (94°C) for 30 s, annealing (56–61°C) for 30 s, and extension (72°C) for 50 s, and ended with a final extension step at 72°C for 8 min.

### Bioinformatics Analysis

Different bioinformatics softwares including Mutation Taster (http://www.mutationtaster.org/), Polyphen-2 (http://genetics.bwh.harvard.edu/pph2), and Sorting Intolerant From Tolerant (SIFT, http://provean.jcvi.org/index.php), were used for functional effect prediction. Finally, for the interpretation of variants, the American College of Medical Genetics and Genomics (ACMG) 2015 guidelines were used.

## Results

### Detailed Clinical Features of the Patient

#### Family 1

**Figure 1:**
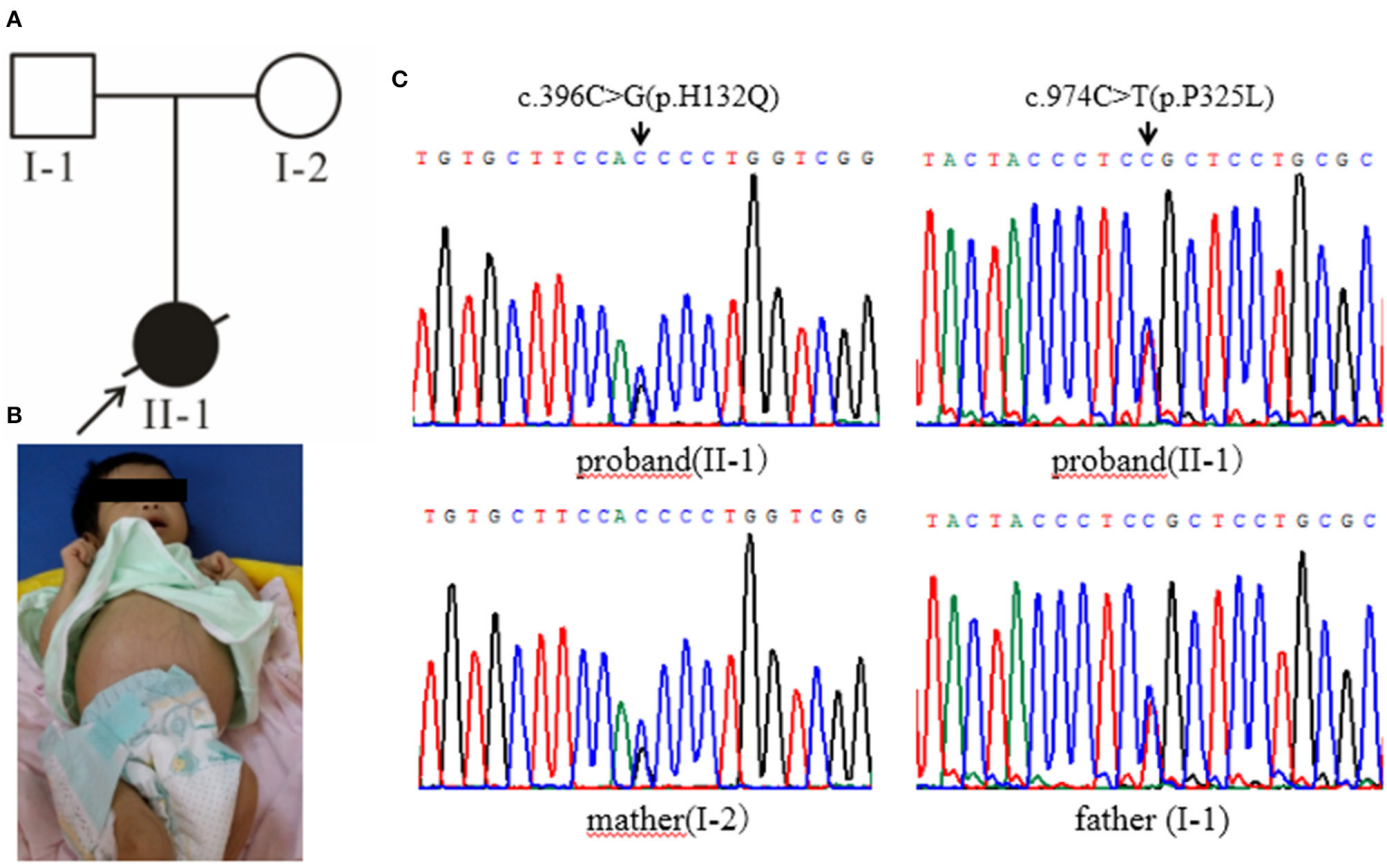
**(A)** The black arrow indicates the proband, and the slash indicates the proband has died. **(B)** The clinical manifestations of the proband: yellow skin, dark yellow color, abdominal distention, and abdominal wall vein filling. **(C)** Gene sequencing results: the proband carries the GALT gene c.396C>G (p.His132Gln) and c.974C>T (p.Pro325Leu) complex heterozygous mutation, c.396C>G (p.His132Gln) from the maternal source, and c.974C>T (p.Pro325Leu) from the paternal source.

Case 1 (Figure 1A, II-1) female subject, 15 days old at the initial diagnosis, was admitted to the Department of Newborn Screening Center, Beijing Obstetrics and Gynecology Hospital, Capital Medical University for reexamination of “increased blood phenylalanine level in neonatal screening.” She was a full-term normal baby, and her mother (gravidity 1, parity 1) experienced a smooth pregnancy this time. For the baby, symptoms of jaundice occurred and recurred in 3rd and 10th day of life. The physical and mental reaction were minor, the full body skin was dark yellow, abdominal distention, abdominal wall vein filling (Figure 1B), hepatomegaly, spleen was not touched. Laboratory examinations showed elevated liver enzymes, total bilirubin, mainly direct bilirubin, accompanied by elevated bile acids (Supplementary Table 1); AFP > 1,000 μg/L; abnormal coagulation function (Supplementary Table 2); serum arginine, citrulline, and tyrosine increased to varying degrees (Supplementary Table 3); urine lactic acid, phenyllactic acid, 4-hydroxyphenyllactic acid increased. Abdominal B-ultrasound showed that: 3.3 cm below the liver ribs and 2.9 cm under the sword, the echo of liver parenchyma was enhanced, the sheath of grissen's was thickened, free ascites was found in the abdominal cavity, the depth was about 4.6 cm; Brain MRI showed that—the echo of brain white matter was slightly strong, and the ventricles were not expanded. The child died after 2 months—due to severe liver failure. There was no family history was seen of Classical Galactosemia.

#### Family 2

**Figure 2:**
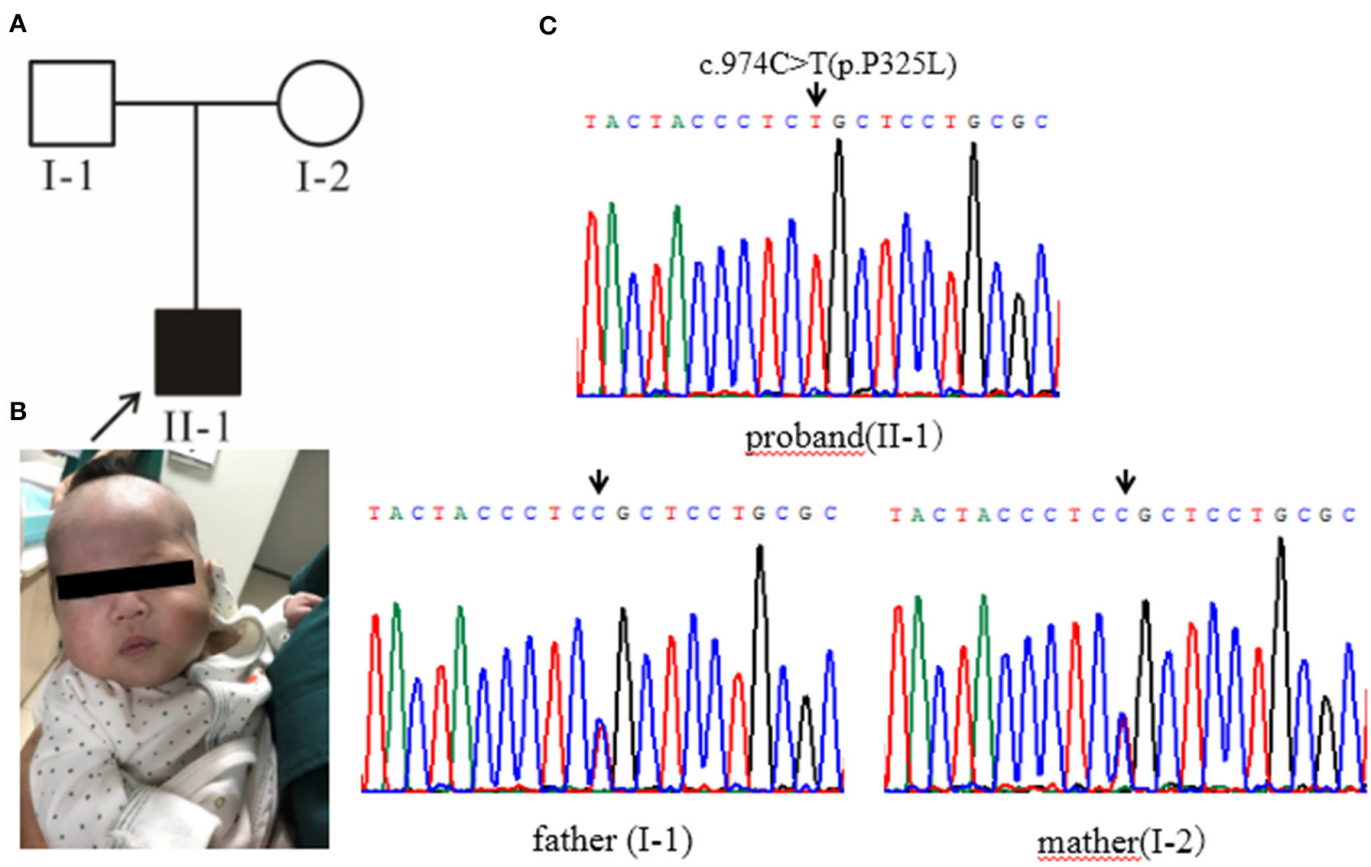
**(A)** Pedigree chart, black arrow indicates the proband. **(B)** The proband's condition improves after being fed milk powder without lactose. **(C)** Gene sequencing results: the proband of pedigree 2 carries the homozygous mutation of GALT gene c.974C>T (p.Pro325Leu) and the parents are all carriers of the mutation.

Case 2 (Figure 2A, II-1): female subject, 12 days old at the initial diagnosis, who was hospitalized in the department of Neonatology, Hebei Provincial Children's Hospital for half a day due to “less milk intake and fever.” She was a full-term normal baby, birth weight 3,200 g and her mother (gravidity 2, parity 2) experienced a smooth pregnancy this time. After the birth of breast-feeding, early self-feeding was good, and then gradually reduced the amount of milk, yellow paste stool, and frequency. Twelve days after birth (half-day before admission) after contact with fever patients (the mother of the child), with the maximum temperature of 38.0°C. The yellow skin stain was found on the 3rd day of birth, but it did not subside. The body weight was 2,970 g (230 g lower than birth), the face was painful, the skin was sallow and flowery, the abdomen was swollen, the veins of the abdominal wall were full, 6.0 cm under the liver rib, 6.0 cm under the sword, 4.0 cm under the spleen rib, soft. In the laboratory, The total bilirubin was significantly increased, mainly the direct bilirubin and the bile acid (Supplementary Table 1); the coagulation function was abnormal (Supplementary Table 2); there was no obvious abnormality in the blood metabolism screening, and urine 4-hydroxyphenyllactate increased; the blood culture was positive for *Escherichia coli*. B-ultrasonography of abdomen showed that the echo of liver parenchyma was enhanced and thickened, the point-like strong echo in the wall of the intestinal tract, and the peritoneal effusion; brain MRI showed that left intracerebral hemorrhage with ventricular enlargement. After admission, the patient was given anti infection and symptomatic treatment. The general condition improved, but there were still gastrointestinal symptoms with poor weight growth. Considering the presence of genetic metabolic diseases, the patient was finally diagnosed by genetic testing. After 3 months of birth, the follow-up showed that there was no vomiting and diarrhea after feeding milk powder without lactose, the mental reaction was good (Figure 2B), the liver function was obviously improved. The parents married without close relatives, there was no family history was seen of Classical Galactosemia.

### Molecular Analysis

High-throughput sequencing data were processed to identify pathogenic mutations in the CG families. Proband from both families (1 and 2) and their parents were selected for high-throughput sequencing. After data analysis and filtrations, homozygous or compound heterozygous non-synonymous variants were selected having MAF >0.1% in different databases including dbSNP, ExAC, and gnomAD, etc. An identified mutation was further verified by Sanger sequencing using ABI3730 Automated Sequencer (PE Biosystems, Foster City, CA). We identified compound heterozygous mutation (c.396C>G; p.His132Gln and c.974C>T; p.Pro325Leu) in family 1 (Figure 1C) and a homozygous missense mutation (c.974C>T; p.Pro325Leu) in family 2 (Figure 2C) respectively. Finally, the identified variants were confirmed by different bioinformatics tools such as Polymorphism Phenotyping v2 (Polyphen-2), Sorting Intolerant from Tolerant (SIFT) and Mutation Taster (Table 1). The prediction results were also supported by the extremely low allele frequencies of the two mutations (Table 1).

**Table 1:** Bioinformatic analysis of *GALT* variants.

	**ExACALL**	**1,000 g 2015 Aug all**	**GnomAD ALL**	**SIFT score**	**SIFT pred**	**Polyphen2 score**	**Polyphen2 pred**	**Mutation Taster score**	**Mutation Taster pred**
c.396C>G (p.H132Q)	–	–	–	0.028	D	0.991	D	1	D
c.974C>T (p.P325L)	–	0.000008237	0.00003232	0	D	1	D	1	D

*D, Damaging; P, Possibly Pathogenic*.

## Discussion

Classic Galactosemia (CG) is a rare autosomal recessive disorder of galactose metabolism. It caused by mutations in the galactose-1-phosphate uridyl transferase (GALT) gene located on chromosome 9p13.3 and has a total length of 4.3kb (7). GALT consists of 11 exons and encodes 379 amino acids expressed highly in liver, red blood cells (RBCs), and other tissues of the body. CG disease caused by the inability to digest galactose, is life-threatening but its pathophysiology has not been clearly defined.

Depending on the enzyme that is deficient (GALK, GALT, or GALE), there are three types of diseases (8). Classic Galactosemia type 1 represents the most severe form of the disease which is caused by the deficiency of GALT. Most of the children at perinatal period, presenting with vomiting, diarrhea, drowsiness, and other neurological symptoms, followed by jaundice, hepatomegaly, hypoglycemia, renal dysfunction, coagulation, and other abnormalities (9, 10). If not treated on time, the symptoms will be aggravated, and finally, death may occur (11, 12). Type II and type III are rare forms of galactosemia caused by GALK and Gale deficiency, respectively. The main clinical feature associate with Type II galactosemia is cataract (1). Due to the poor specificity of the clinical manifestations of gale patients, it is difficult to diagnose the disease. Therefore, it is very important for the rapid diagnosis and treatment of the disease to explore the genetic causes of gale patients through high-throughput sequencing technology, combined with conventional differential diagnosis. GALT gene presents high allelic heterogeneity and more than 100 mutations have been identified in human gene mutation database (HGMD: http://www.hgmd.org). The most common mutations in various populations identified in GALT are the Gln188Arg, Lys 285Asn, Ser135Leu, and Asn314Asp (13–15).

Here, we report two families with CG who were detected during a neonatal screening in China. Clinical investigation of family 1 showed full abdominal vein, hepatomegaly (Figure 1B), elevated level of serum arginine, methionine, citrulline, and tyrosine (Supplementary Table 3), and the level of lactate and phenylactate in urine were increased. In family 2, there were symptoms including fever, hepatosplenomegaly, intrauterine infection, and hematologic disorders. Molecular study of both families (1 and 2) revealed compound heterozygous (c.396C>G; p.His132Gln and c.974C>T; p.Pro325Leu) (Figure 1C) and homozygous missense (c.974C>T; p.Pro325Leu) (Figure 2C) mutations in GALT gene in family 1 and 2 respectively. Through molecular simulation prediction, it is suggested that p.His132Gln indirectly destroys the active site of the enzyme, decrease GALT enzymatic activity, and finally effects protein translation and /or protein stability (16). Although the mutation c.396C>G has not been previously reported, it leads to the same amino acid substitution (p.His132Gln) as the mutation c.396C>A which has been previously reported and included in the Clinvar database (16). Given that the effects of this substitution has been well-studied, it is reasonable to determine the pathogenicity of this missense mutation. The homozygous missense mutation c.974C>T; p.Pro325Leu changes the proline to leucine at 325th amino acid lead to CG phenotypes. *In silico* analysis, it is predicted that the mutations were pathogenic that lead to CG. Although c.974C>T (p. p.Pro325Leu) is a known mutation (17), but it is the first found in the Chinese population. This study verifies the pathogenicity of the mutation again. The clinically features observed in our patients were jaundice and hepatomegaly, which accorded with the typical characteristics of galactosemia in infancy (6).

The conventional differential diagnosis methods of gal are enzymatic diagnosis and chemical diagnosis (8, 13). Enzymology diagnosis is easy to be interfered by external environmental factors, temperature, and humidity are too high to cause false negative or false positive. Chemical diagnosis can only get positive results when the disease attacks and is greatly affected by diet, which can only be used as a means of screening, not as a basis for diagnosis. At the same time, the study found that neonatal intrahepatic cholestasis caused by citrin deficiency (NICCD) is very similar to galactosemia in clinical manifestations and experimental examination, but there is a large difference in later treatment (14, 15). Meanwhile, the case 2, began to “fever” treatment, for hepatosplenomegaly, initially suspected of intrauterine infection, blood system disease, hemophagocytic syndrome, or congenital genetic metabolic disease? After a series of clinical examinations, no diagnosis was made. Therefore, it is very important to find an accurate and effective clinical differential diagnosis method for patients' prognosis, clinical treatment, and genetic consultation. With the rapid development of high-throughput sequencing technology, the target gene can be accurately captured and the disease species with high genetic heterogeneity can be comprehensively analyzed (18). Not only the operation cost is low, the flux is high, and the operation speed is fast, but also the results are reliable and stable. At present, it has been successfully applied to the diagnosis of multiple genetic metabolic diseases such as methylmalonic acidemia, maple syrup uremia, ornithine carbamylase deficiency, etc. (19).

In conclusion, use of high-throughput sequencing technology, can identify the cause of disease and improve the efficiency of disease diagnosis. In particular, we should pay more attention to gene detection in children with persistent jaundice and abnormal liver function. Achieving early detection early diagnosis and early treatment can provide effective genetic consultation and prenatal diagnosis for patients' families.

## Data Availability Statement

The raw data supporting the conclusions of this article will be made available by the authors, without undue reservation.

## Ethics Statement

The studies involving human participants were reviewed and approved by Ethics Committee of Beijing Obstetrics and Gynecology Hospital affiliated to Capital Medical University. Written informed consent to participate in this study was provided by the participants' legal guardian/next of kin. Written informed consent was obtained from the individual(s), and minor(s)' legal guardian/next of kin, for the publication of any potentially identifiable images or data included in this article.

## Author Contributions

LL and LM performed the sequencing analysis, and wrote the manuscript. MS, JJ, and YZ conducted data collection as well as data analysis. YT and NY helped with recruiting patients and YT helped to discuss the data. All authors performed critical reading and approved the final version of manuscript. YK conceived the study and supervised this research.

## Conflict of Interest

The authors declare that the research was conducted in the absence of any commercial or financial relationships that could be construed as a potential conflict of interest.

## References

[1] Demirbas D Coelho AI Rubio-Gozalbo ME Berry GT. Hereditary galactosemia. Metabolism. (2018) 83:188–96. 10.1016/j.metabol.2018.01.025 29409891

[2] Calderon FR Phansalkar AR Crockett DK Miller M Mao R. Mutation database for the galactose-1-phosphate uridyltransferase (GALT) gene. Hum Mutat. (2007) 28:939–43. 10.1002/humu.20544 17486650

[3] Holden HM Rayment I Thoden JB. Structure and function of enzymes of the leloir pathway for galactose metabolism. J Biol Chem. (2003) 278:43885–8. 10.1074/jbc.R300025200 12923184

[4] Garcia DF Camelo JS Jr Molfetta GA Turcato M Souza CFM Porta G. Clinical profile and molecular characterization of galactosemia in brazil: identification of seven novel mutations. BMC Med Genet. (2016) 17:39. 10.1186/s12881-016-0300-8 27176039 PMC4866286

[5] Choi R Jo KI Ko D-H Lee DH Song J Jin D-K. Novel GALT variations and mutation spectrum in the Korean population with decreased galactose-1-phosphate uridyltransferase activity. BMC Med Genet. (2014) 15:94. 10.1186/s12881-014-0094-5 25124065 PMC4236512

[6] Timson DJ. The molecular basis of galactosemia - past, present and future. Gene. (2016) 589:133–141. 10.1016/j.gene.2015.06.077 26143117

[7] De Lucca M Barba C Casique L. A novel splicing mutation in GALT gene causing Galactosemia in Ecuadorian family. Clin Chim Acta. (2017) 470:20–3. 10.1016/j.cca.2017.04.021 28450132

[8] Holton JB Walter JH Tyfield LA. The Metabolic and Molecular Basis of Inherited Disease. New York: McGraw-Hill (2001).

[9] Welling L Boelen A Derks TG Schielen PC de Vries M Williams M. Nine years of newborn screening for classical galactosemia in the Netherlands: effectiveness of screening methods, and identification of patients with previously unreported phenotypes. Mol Genet Metab. (2017) 120:223–8. 10.1016/j.ymgme.2016.12.012 28065439

[10] Bosch AM. Classical galactosaemia revisited. J Inherit Metab Dis. (2006) 29:516–25. 10.1007/s10545-006-0382-0 16838075

[11] Holton JB. Galactosaemia: pathogenesis and treatment. J Inherit Metab Dis. (1996) 19:3–7. 10.1007/BF01799341 8830174

[12] Potter NL Nievergelt Y Shriberg LD. Motor and speech disorders in classic galactosemia. JIMD Rep. (2013) 11:31–41. 10.1007/8904_2013_219 23546812 PMC3755563

[13] Viggiano E Marabotti A Burlina AP Cazzorla C D'Apice MR Giordano L. Clinical and molecular spectra in galactosemic patients from neonatal screening in northeastern Italy: Structural and functional characterization of new variations in the galactose-1-phosphate uridyltransferase (GALT) gene. Gene. (2015) 559:112–8. 10.1016/j.gene.2015.01.013 25592817

[14] Seyrantepe V Ozguc M Coskun T Ozalp I Reichardt JK. Identification of mutations in the galactose-1-phosphate uridyltransferase (GALT) gene in 16 Turkish patients with galactosemia, including a novel mutation of F294Y. Hum Mutat. (1999) 13:339. 10.1002/(SICI)1098-1004(1999)13:4<339::AID-HUMU17>3.0.CO;2-V 10220154

[15] Schulpis K Papakonstantinou ED Michelakakis H Podskarbi T Patsouras A Shin Y. Screening for galactosemia in Greece. Paediatr Perinat Epidemiol. (1997) 11:436–440. 10.1046/j.1365-3016.1997.d01-31.x 9373865

[16] Tang M Facchiano A Rachamadugu R Calderon F Mao R Milanesi L. Correlation assessment among clinical phenotypes, expression analysis and molecular modeling of 14 novel variations in the human galactose-1-phosphate uridylyltransferase gene. Hum Mutat. (2012) 33:1107–15. 10.1002/humu.22093 22461411 PMC3431212

[17] Greber-Platzer S Guldberg P Scheibenreiter S Item C Schuller E Patel N. Molecular heterogeneity of classical and duarte galactosemia: mutation analysis by denaturing gradient gel electrophoresis. Hum Mutat. (1997) 10:49–57. 10.1002/(SICI)1098-1004(1997)10:1<49::AID-HUMU7>3.0.CO;2-H 9222760

[18] Li L Zhao J-Q Wang C Yang N Gong L-F Yang H-H. Whole-exome sequencing as a powerful tool for identifying genetic causes in a patient with POLG-related disorders and phenylketonuria. J Int Med Res. (2019) 47:1387–94. 10.1177/0300060518823096 30678510 PMC6421386

[19] Tong W Wang Y Lu Y Ye T Song C Xu Y. Whole-exome sequencing helps the diagnosis and treatment in children with neurodevelopmental delay accompanied unexplained dyspnea. Sci Rep. (2018) 8:5214. 10.1038/s41598-018-23503-2 29581464 PMC5980106

