# Supplementary material for: High-Throughput Sequencing Reveals the Loss-of-Function Mutations in GALT Cause Recessive Classical Galactosemia
Source: Front Pediatr. 2020 Aug 5;8:443. doi: 10.3389/fped.2020.00443 (PMC7438714; doi:10.3389/fped.2020.00443)
Supplement: Supplementary file 1 [file Data_Sheet_1.PDF]

Supplementary Table 1 Test results of biochemical index

Detection index	case 1	case 2	reference value
Total bilirubin (umol/L)	190.00 ↑	239.00 ↑	3.40-17.00
Direct bilirubin (umol/L)	114.70 ↑	199.90 ↑	0.00-3.40
Alanine aminotransferase (IU/L)	75.00 ↑	116.00 ↑	5.00-40.00
Aspartate aminotransferase (IU/L)	139.00 ↑	140.00 ↑	5.00-40.00
Lactate dehydrogenase (IU/L)	328.00 ↑	-	109.00-245.00
Glutamyltranspeptidase (IU/L)	15.00	42.00	7.00-30.00
Total protein quantification (g/L)	40.90 ↓	-	60.00-83.00
Albumin quantification (g/L)	19.20 ↓	-	34.00-54.00
Serum total bile acid (umol/L)	112.00 ↑	297.50 ↑	0.00-10.00

Supplementary Table 2 Test results of coagulation function

Detection index	case 1	case 2	reference value
Prothrombin time(sec)	23.4 ↑	18.5↑	9.4-12.5
International standardized ratio	2.04	1.64	——
Fibrinogen(g/L)	1.95	3.16	2-4
Activated partial thromboplastin time(sec)	119.1↑	71.0↑	25.1-38.4

Supplementary Table 3 Test results of blood tandem mass spectrometry

Detection index	case 1	case 2	reference value (umol/L)
PHE	85.7	34.28	30-110
PHE/TYR	0.18	1.51 ↑	0.1-1.5
TYR	482.57 ↑	22.69 ↓	40-400
SA	0.52	1.03 ↑	0.3-1.00
MET	51.5	14.36	10-60
MET/PHE	0.60	0.42	0.2-1.1
VAL	234.3	62.13	60-280
LEU+Ile+Pro-OH	218	41.2 ↓	90-350
(LEU+Ile+Pro-OH)/PHE	2.54	1.20 ↓	1.5-6
VAL/PHE	2.73	1.81	1.0-4.8
CIT	118.38 ↑	18.33	7.6-50

CIT/PHE	1.38 ↑	0.53	0.1-1.0
CIT/ARG	1.21	2.17	0.3-10.0
ARG	97.8 ↑	8.43	2.0-60
ORN	341.16	73.52	60-400
ARG/PHE	1.14 ↑	0.25	0.03-1.3
ORN/PHE	3.98	2.14	1-6
ALA	392.85	158.42 ↓	170-740
GLY	857.51	373.42	210-1200
GLY/ALA	2.18	2.35	0.6-3
PRO	455.58	95.39 ↓	110-480
